# Spontaneous Recovery of Inferior Oblique Overaction in Three Saudi Children: A Case Report

**DOI:** 10.7759/cureus.51152

**Published:** 2023-12-27

**Authors:** Dora H AlHarkan

**Affiliations:** 1 Ophthalmology, Qassim University, Buridah, SAU

**Keywords:** extraocular muscle surgery, extraocular muscle, pediatrics ophthalmology, inferior oblique overaction, strabismus

## Abstract

The inferior oblique muscle overaction (IOOA) results in eye elevation on adduction, head tilt, difficulty in reading/writing, and changing ocular alignments in different gazes. Surgical correction is the management. We present two cases of bilateral and one case of unilateral IOOA that resolved spontaneously, and surgery differed. There was no IOOA six months after diagnosis. A sustained resolution IOOA following correction of hyperopia, improvement of vision, and esotropia correction possibly leading to rebalancing and maturing of extraocular muscles. Ophthalmologists should refer all IOOA cases to strabismologists, should not hurry to operate, counsel parents, and monitor a child’s ocular movements.

## Introduction

The inferior oblique muscle is an extraocular muscle. It is attached to the maxillary bone near the lacrimal fossa and inserted into the posterior part of the eye, inferior to the insertion of the lateral rectus after passing under the belly of the inferior rectus. The inferior oblique muscle is innervated by the lower portion of the oculomotor nerve (cranial nerve III) [1].

Inferior oblique muscle overaction (IOOA) is an ocular motility anomaly consisting of overelevation during adduction and is often associated with ipsilateral hypertropia. Primary IOOA is usually bilateral but can be asymmetric and can present with elevation of the eye upon adduction, slight vertical deviation in the primary position, minimal head tilt, or a negative Bielschowsky test [2]. Secondary IOOA is usually unilateral and can be secondary to paresis or paralysis of the ipsilateral superior oblique muscle [3].

Primary IOOA is present in almost 70% of patients with infantile esotropia, 35% of patients with accommodative esotropia, and 30% of patients with exotropia [4]. Surgical management is indicated if IOOA is severe and affects near-work activities such as studying (writing and reading) or climbing stairs. Surgical treatment options include recession, anteriorization, or myectomy [5]. Anti-elevation, hypotropia, recurrence, and fat adherence syndrome (13%) are the most common complications of IOOA surgeries [6]. Parents often delay or deny surgery for IOOA in their child because the primary position of gaze is not shown or affected. Therefore, additional justification is needed for performing surgery for IOOA.

According to the literature, only a few cases of spontaneous recovery from IOOA have been reported, and these cases occurred just after surgical intervention for esotropia but not after spontaneous resolution without surgical intervention [7,8]. We present three children with IOOA that resolved spontaneously without any surgery.

## Case presentation

Case 1

**Figure 1:**
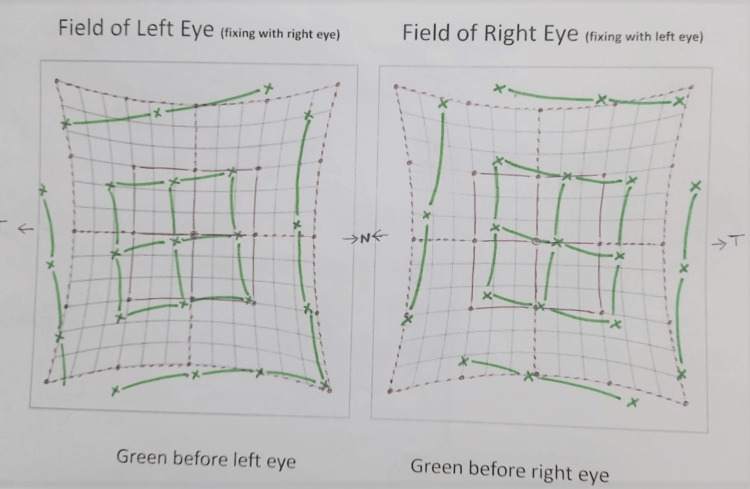
Hess chart showing ocular movements of a child with bilateral inferior oblique muscle overaction. The red lines depict normal muscle movements at different gazes, while the green lines depict the extraocular muscle action of the present patient at different gazes. The central green dot deviates on the temporal side more than the central green dot, suggesting esotropia. The outer green lines cross the normal red lines and are not uniting in the supranasal area of either eye. The inner green lines are directed toward the supranasal area in both eyes, suggesting inferior oblique overaction.

This was a case of fully accommodative esotropia with mild amblyopia in the left eye and bilateral IOOA. The patient presented for the first time at the age of 5 years, with a complaint of inward deviation of the left eye starting at the age of two and a half years. He wore glasses, and his best corrected visual acuity (BCVA) was 20/30 in the right eye and 20/80 in the left eye. The extraocular muscle movement (EOM) was normal in all eight cardinal directions of gaze, with +3 inferior oblique overaction (IOOA) in both eyes. He had left esotropia of 8 prism diopters (PDs) on the fixing object for near with correction (NCC). On fixing the object for distance with correction (DCC), he had left esotropia of 6 PD. He had left eye esotropia of 16 PD on near fixation without correction (NSC) and esotropia of 14 PD while fixating on a distant object without correction (DSC). He had signs and symptoms of allergic conjunctivitis with inferior exposure keratopathy. His cycloplegia in the right eye was +4.50 D (spherical) and +3.00 D (cylindrical) on the 95º axis. In the left eye, the refraction was +4.75 D (spherical) and +2.50 D (cylindrical) at the 70º axis. He was given spectacles and was instructed to have part-time occlusion of the right eye. No intervention was performed for the IOOA because it did not affect the patient’s life. At the one-year follow-up, when the child was six years old, his vision, esotropia, and amblyopia improved, and he was wearing proper glasses and patching for amblyopia treatment. His best-corrected vision was 20/20 in the right eye and 20/25 in the left eye. Examination of the EOM showed a right of IOOA +3 and a left IOOA of +3. By orthoptic work-up, it was shown that he was orthotropic both near and distance. He started attending his first year of elementary schooling, and the mother complained that the child had a head tilt while focusing on books and had difficulty writing and reading. Therefore, he was offered bilateral inferior oblique recession surgery, but his parents were hesitant to do so. His Hess chart is given in Figure 1.

**Figure 2:**
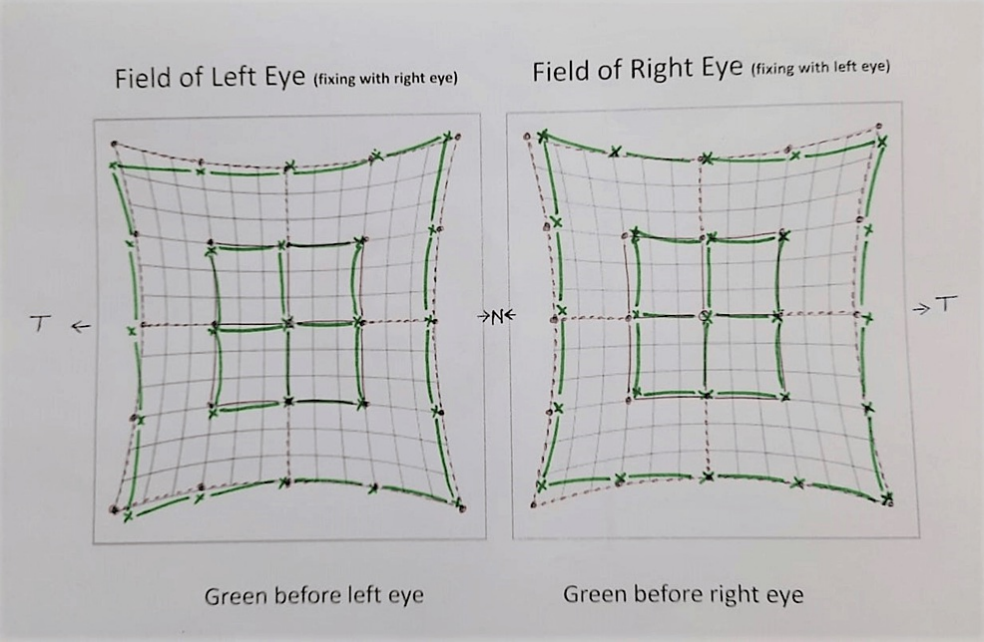
Hess chart of the same child showing ocular movements after spontaneous resolution of inferior oblique muscle overaction. Green lines (present patients) indicate muscle movements with different gaze overlaps; red lines (normal muscle movements) suggest resolution of the inferior oblique overaction.

The mother was assured of the child’s symptoms of head tilt, and the mother was told that if the child’s school performance worsens, the child is encouraged to undergo surgery. Later, when the child was seven years old, he came for a follow-up. On examination, we noted that there was no additional inferior oblique overaction, and the mother reported that the head tilt was resolved and that the child was doing better in writing and reading. The last follow-up was performed in 2023, and there was no evidence of IOOA. The Hess chart at this follow-up is shown in Figure 2.

Case 2

This was a case of hyperopia with asthenopia and unilateral significant IOOA. A five-year-old girl presented with complaints of difficulty writing and reading. Her UCVA for distance in both eyes was 20/25. She had orthophoria. The EOM was normal in all directions, but she had a +3 IOOA in the left eye. Her cycloplegia in the right eye was +2.00 D (spherical) and +0.75 D (cylindrical) at the 95º axis. In the left eye, the refraction was +2.00 D (spherical) and +0.50 D (cylindrical) at the 85º axis. She was prescribed spectacles with +0.50D spherical and +0.75D cylindrical at the 95º axis to the right eye and in the left eye and +0.50D spherical and +0.50D cylindrical at the 85º axis. Her BCVA at two months after management was 20/20 in both eyes. One year later, on examination, when she was six years old, her BCVA was 20/20, and there was no IOOA.

Case 3

This is a case of accommodative esotropia with bilateral asymmetric IOOA. The child was brought by his parents when he was seven years old and had complaints of crossing eyes and poor focusing and writing in school. His BCVA was 20/20 in the right eye and 20/25 in the left eye. His EOM was normal, but his right IOOA was grade +1, and his left IOOA was grade +3. NCC and DCC were left-sided esotropia with a 12ΔD. The cycloplegia refraction parameters were +2.75D sph and +0.50D cylindrical on-axis 45º in the right eye and +3.00D sph and +0.50D cyl axis 120º in the left eye. He was happy with his vision and corrected esotropia but had problems with his performance in school. He was therefore advised to undergo bilateral inferior oblique recession surgery for IOOA, but he was hesitant to undergo surgery. The child was lost to follow-up. After one year, when he reported that although his performance in school improved, he was convinced of the need for IOOA surgery. On examination before surgery, we found that his IOOA had resolved. Therefore, his surgery was canceled, and he was advised to continue follow-up.

## Discussion

Two patients had bilateral IOOA, and one had unilateral IOOA. Of them, two had esotropia, and one had orthophoria. All three patients were hyperopic and did not have any associated known syndrome known to be associated with esotropia or IOOA. In the two patients with esotropia, the esotropia was fully accommodative and started after the age of two years. In cases of esotropia, the presentation of IOOA resolved spontaneously with proper treatment for esotropia, spectacle correction, and improvement of amblyopia. The resolution was within the age range of six to seven years. The IOOA did not recur in the eyes, and two of them had a follow-up of more than one year.

Spontaneous resolution or recovery of the affected extraocular muscles has been documented in the literature. However, this recovery occurs mainly in the eyes following nerve paresis or trauma to the ocular muscle during ocular surgery. A study was performed on 28 patients to evaluate the status of IOOA after surgical correction of esotropia. Of those, 79% had infantile esotropia, and 21% had partially accommodative esotropia. IOOA regressed without touching the inferior oblique muscle in almost 81% of patients and completely resolved in almost 36% of them [8]. In that report, IOOA was resolved after management and correction of esotropia. However, in two of our patients, the IOOA resolved without surgical intervention for esotropia. It is therefore recommended that, before patients proceed with surgery for any inferior oblique overaction, proper management of esotropia should be performed either with full-power correction of hyperopia or with surgical intervention for residual esotropia and with proper treatment of amblyopia. Then, the patient should be evaluated for at least six months to one year of follow-up to provide time for possible spontaneous resolution of IOOA [7].

In a study performed by Stager et al., elevated collagen and elastin levels were found in the surgical specimens of patients with primary inferior oblique overaction compared with the control group [9]. The surgical intervention resulted in a normal connective tissue profile of IOOA muscles in 16 eyes with IOOA. In another study, elevated numbers of neuromuscular junctions relative to the whole muscle area were noted in muscles with IOOA [10]. It is postulated that primary oblique muscle overaction could be due to early-onset strabismus or structural lesions within the posterior fossa affecting central vestibular tone [11]. It seems that the connective tissue matrix is more abnormal in eyes with IOOA than in normal muscles or muscles after surgery. Perhaps these muscles with overaction mature with age, resulting in spontaneous resolution.

Our study cautions pediatric ophthalmologists about not being in a hurry about the surgical management of IOOA, especially for IOOA patients associated with esotropia. Adequate time should be given for IOOAs to resolve after correcting esotropia with glasses or by surgical intervention (managing the horizontal component of muscle imbalance) and after improving amblyopia in such children. By avoiding surgeries to the inferior oblique, we can decrease the risk of complications such as fat adherence syndrome, recurrence, or hypotropia. Although parents should not be given a promise of resolution, they could be counseled on providing examples of spontaneous resolution, which would also improve compliance with spectacle wearing in children and periodic follow-up to determine muscle status. In addition to crossing eyes, poor performance at school and abnormal head posture are some of the main symptoms of children with ocular muscle anomalies, and teachers should be made aware of these indicators and the need for ophthalmic reference in such children.

A comprehensive workup of a patient with IOOA, including 9-gaze EOM photos with spectacle correction, is suggested for follow-up. Two of our patients were booked for surgery, but unfortunately, no photos were taken before the resolution of the IOOAs. Unfortunately, we did not have photographs at the presentation for our patients. Luckily, we had a Hess chart for one patient demonstrating IOOA before and after resolution. However, in young children, it is difficult to do an MRI of the IO muscle, which will help in understanding the anatomical variation of the IO in patients with IOOA [12].

## Conclusions

These cases of spontaneous resolution of IOOA affecting ocular health and scholastic performance in children warn ophthalmologists not to hurriedly plan surgeries and work with parents to monitor the progress of their ocular muscle status after addressing underlying ailments such as hyperopia, esotropia, and amblyopia.
